# GlycoFibroTyper: A Novel Method for the Glycan Analysis of IgG and the Development of a Biomarker Signature of Liver Fibrosis

**DOI:** 10.3389/fimmu.2022.797460

**Published:** 2022-02-07

**Authors:** Danielle A. Scott, Mengjun Wang, Stephane Grauzam, Sarah Pippin, Alyson Black, Peggi M. Angel, Richard R. Drake, Stephen Castellino, Yuko Kono, Don C. Rockey, Anand S. Mehta

**Affiliations:** ^1^ Glycopath, Inc., Charleston, SC, United States; ^2^ Department of Cell and Molecular Pharmacology & Experimental Therapeutics, Medical University of South Carolina, Charleston, SC, United States; ^3^ Department of Medicine, Gastroenterology and Hepatology, University of California San Diego, San Diego, CA, United States; ^4^ Digestive Disease Research Center, Medical University of South Carolina, Charleston, SC, United States

**Keywords:** glycosylation, immunoglobulin, fibrosis, cirrhosis, biomarker

## Abstract

Our group has recently developed the GlycoTyper assay which is a streamlined antibody capture slide array approach to directly profile N-glycans of captured serum glycoproteins including immunoglobulin G (IgG). This method needs only a few microliters of serum and utilizes a simplified processing protocol that requires no purification or sugar modifications prior to analysis. In this method, antibody captured glycoproteins are treated with peptide N-glycosidase F (PNGase F) to release N-glycans for detection by MALDI imaging mass spectrometry (IMS). As alterations in N-linked glycans have been reported for IgG from large patient cohorts with fibrosis and cirrhosis, we utilized this novel method to examine the glycosylation of total IgG, as well as IgG1, IgG2, IgG3 and IgG4, which have never been examined before, in a cohort of 106 patients with biopsy confirmed liver fibrosis. Patients were classified as either having no evidence of fibrosis (41 patients with no liver disease or stage 0 fibrosis), early stage fibrosis (10 METAVIR stage 1 and 18 METAVIR stage 2) or late stage fibrosis (6 patients with METAVIR stage 3 fibrosis and 37 patients with METAVIR stage 4 fibrosis (cirrhosis)). Several major alterations in glycosylation were observed that classify patients as having no fibrosis (sensitivity of 92% and a specificity of 90%), early fibrosis (sensitivity of 84% with 90% specificity) or significant fibrosis (sensitivity of 94% with 90% specificity).

## Introduction

Cirrhosis is the result of chronic liver injury and leads to replacement of the normal liver architecture by fibrotic scar tissue, and is associated with a concomitant decline of liver function and devastating clinical complications (1). Many different underlying processes cause cirrhosis: chronic viral infection by hepatitis B virus (HBV) and/or hepatitis C virus (HCV) have been historically among the most common etiologies, but non-alcoholic fatty liver disease and ethanol are currently emerging as the leading causes of chronic liver disease worldwide.

For HBV and/or HCV infected patients, treatment decisions are based upon a variety of factors, including elevated levels of hepatic transaminases which may reflect the degree of hepatic inflammation and perhaps fibrosis when combined with other features such as platelet count (2, 3). Historically, in individuals with HBV or HCV, advanced fibrosis and cirrhosis are considered justifications to begin antiviral therapy (2, 4, 5). More importantly, the determination of hepatic fibrosis is critical to stage the severity of the liver disease and has been associated with prognosis (6). Therefore, it is extremely important to determine the presence of significant fibrosis and cirrhosis. Although liver biopsy has been historically the gold standard for assessment of fibrosis (7) noninvasive assessment of fibrosis is less intrusive and will allow for routine clinical monitoring.

Analysis of disease associated changes in total N-glycan compositions of serum and plasma in large patient cohorts has been previously reported (8, 9). There has also been extensive evaluation of disease-associated alterations of immunoglobin G N-linked glycans in large sample cohorts in patients with rheumatoid arthritis, digestive diseases, heart disease, cancer and liver fibrosis (10–34). In the case of liver fibrosis, two analytical methods have identified alterations in N-linked glycosylation on both total IgG populations and on specific IgG molecules. Both methods can detect cirrhosis with a high degree of accuracy and are also able to detect intermediate levels of fibrosis (11, 16). However, both analytical methods have drawbacks. One approach utilizes a capillary electrophoresis-based analysis of N-linked glycans on total serum following release of glycans using PNGase F, labeling of the released glycans using a fluorescent dye, and electrophoresis of the released glycans for peak identification (11). While this method works well, it is both time and labor intensive. Another method for liver fibrosis identification from serum utilizes a plate-based ELISA format to detect altered glycosylation using a sugar binding protein called a lectin (16). This test is hampered by the poor specificity of lectins and the limited readout (i.e. binding or not). Both of these methods require specialized sample handling resources, extensive processing and purification prior to analysis, and are expensive in regard to reagents and processing.

Our group has recently developed the GlycoTyper, a streamlined antibody capture slide array approach to directly profile N-glycans of captured serum glycoproteins like IgG. The method needs only a few microliters of serum and utilizes simplified processing workflows that requires no purification or sugar derivatizations as a part of the analysis. In this method, N-linked glycans are released from antibody captured glycoproteins and are directly analyzed by MALDI IMS, building on the utility of MALDI MS glycan imaging developed in our laboratories (35–48). Here, we have used the GlycoTyper to demonstrate its ability to measure changes in the N-linked glycans on IgG subtypes and serve as a surrogate marker of liver disease.

## Materials and Methods

### Patients

Samples were from two main sources. The first was from the University of California at San Diego and the second from the Medical University of South Carolina. In both cases, the study protocol was approved by the appropriate Institutional Review Board and written informed consent was obtained from each subject. Demographic and clinical information was obtained, and a blood sample was collected from each subject. All liver biopsies were graded using the METAVIR scoring system (49). The etiology of the liver disease for the patients without viral infection was determined as previously described (50) and cirrhosis was determined based on histologic and/or clinical findings (i.e., clinical features of portal hypertension, with no alternative cause of portal hypertension). Details on all patients are provided in **Table 1**. A group of individuals with no history of liver disease, alcohol consumption less than 40 g a week, and no risk factors for viral hepatitis were utilized as controls. All subjects in this control group were documented to have normal liver biochemistry and negative HCV antibodies or HBV.

**Table 1:** Description of control patients and those with liver fibrosis.

		Stage of Fibrosis (METAVIR)^1^				
Variables	Controls	0	1	2	3	4
Sample Size	38	3	10	18	6	37
Age^2^	51 ± 11	56 ± 7	50 ± 6	51 ± 4	51 ± 7	55 ± 8
% NHW/AA/H/Asian^3^	NA	100/0/0/0	98/1/1/0	96/2/1/1	100/0/0/0	98/0/1/0
ALT (IU/mL)^4^	NA	75 ± 7	71 ± 11	78 ± 9	72 ± 12	85 ± 26
AST (IU/mL)^5^	NA	73 ± 5	69 ± 8	77 ± 10	78 ± 13	108 ± 24
Total Bilirubin (mg/dL)	NA	0.3 ± 0.2	0.3 ± 0.4	0.5 ± 0.4	0.9 ± 1.2	1.2 ± 1.3

^1^Fibrosis staging based upon METAVIR scoring system. ^2^Mean age in years. ^3^NHW, non-Hispanic White; AA, African American; H, Hispanic; ^4^ALT, alanine aminotransferase; ^5^AST, aspartate aminotransferase.

NA, not applicable.

### Antibody Array Preparation

The serum assay used a 24-well slide module (Grace -Bio Labs, Bend, OR) that was mounted to a nitrocellulose-coated microscope slides (Grace-Bio Labs, Bend, OR). Antibodies: anti-human (IgG (Bethyl Labs, Montgomery, TX) anti-IgG1 (Abcam, Cambridge, UK), anti-IgG2, anti IgG-3, and anti-IgG 4 (Bio-Rad, Hercules, CA), were diluted in PBS and manually spotted in wells at 200 ng per 1.5 μL spot. Spots were then left to adhere overnight at 4°C in a humidity chamber made from cell culture dishes lined with a Wypall X 60 paper towel and two rolled KimWipes saturated with distilled water. Slides were then placed in a vacuum desiccator to dry at room temperature and rinsed for 1 minute with 200 µL 0.1% octyl-β-D- glucopyranoside in PBS (referred to as PBS-OGS) per well to remove any unbound protein from the slide. More detail on slide preparation and the general GlycoTyper method can be found in (41, 42).

### Sample Capture and N-Glycan Release

Antibody spots were blocked with 200 μL of 2% BSA (prepared in PBS-OGS) per well for 1 h with gentle shaking, washed with 200 μL PBS (3 min × 2) and 200 μL double distilled water (1 min × 1) per well and dried in a desiccator. Pure protein (0.5μg in 100 μL) or serum samples (diluted 1:100 in PBS to a total volume of 100 µL) were added to wells and incubated at room temperature for 2 hours in a humidity chamber with gentle shaking. Pure proteins: human IgG (Jackson ImmunoResearch Laboratories, West Grove, PA), human IgG1, IgG2, IgG3 and IgG4 (Abcam, Cambridge, UK). Wells were then serially washed with 200 μL of PBS-OGS (1 min ×2), 200μL PBS (3 min ×2), 200 μL double distilled water (1 min ×2), and dried. The well module was removed, and slides were dried in a vacuum desiccator. PNGase F Prime PRIME-LY (N-Zyme Scientifics, Doylestown, PA) (0.1 μg/μL in HPLC grade water) was applied to the slides using an automated sprayer (M5 TM-Sprayer, HTX Technologies, Chapel Hill, NC). Spatial localization to each capture spot was accomplished using spraying parameters of 15 passes at 45°C, 10 psi, flow rate of 25 μL/min, and 1200 mm/min velocity. Slides were incubated overnight at 37°C in humidity chambers made in cell culture dishes with Wypall X 60 paper towels and two rolled KimWipes saturated with distilled water.

### MALDI MS Preparation and Imaging

MALDI matrix α-cyano-4-hydroxycinnamic acid (CHCA, 7 mg/mL in 50% acetonitrile/0.1% trifluoroacetic acid) (Cayman Chemical, Ann Arbor, MI) was applied to slides using the same automated sprayer. Matrix was sprayed for two passes at 77°C, 10 psi, 1300 mm/min velocity, and flow rate of 100 μL/min. MALDI IMS data were acquired on a solariX Legacy 7T FT-ICR mass spectrometer (Bruker, Billerica, MA) equipped with a SmartBeam II laser operating at 2000 Hz and pixel dimension of 25 μm. Images were collected using a smartwalk pattern at a 300 μm raster with 200 laser shots per pixel. Samples were analyzed in positive ion with a mass range of 500−5000 m/z using a 512k word time domain. An on-slide resolving power of 58,000 at m/z 1501 was calculated.

### Data Analysis

N-Glycan localization and intensity were visualized using FlexImaging v5.0 (Bruker), with data imported at a 0.98 ICR reduction noise threshold. Images were normalized to total ion current, and N-glycan peaks were selected manually based on their theoretical mass values. For quantification of peaks at individual spots, spectra were imported into SCiLS Lab software 2017a (Bruker). Each spot was designated a unique region, and area under monoisotopic peak values were exported from each region and quantified by mean peak intensity. In all cases, glycan peaks (specific m/z values) were given a relative percentile of the total glycan profile. Glycan names are provided using the Oxford Nomenclature (51).

### Statistical Analysis

Patients were grouped as those: i) without any liver disease (healthy and stage 0), ii) with early to moderate fibrosis (stage 1 &2), and iii) with advanced fibrosis or cirrhosis (stage 3&4). One-way ANOVA and *Post hoc* tests were applied to inference any statistical difference among these three stages. Glycans with no statistical difference among stages (p>0.05, F-test) were discarded from further model development.

Subsequently, we applied the supervised learning algorithm random forest to determine the relative importance of the remaining features (glycans). Simultaneously, we applied variable clustering to perform a hierarchical cluster analysis, which is based on the similarity matrix that contains pairwise Hoeffding D statistics (52). From the variable clustering output, the most highly correlated two features were identified. These two features’ relative importance were derived from random forest analysis. We discarded one of these pairs with lower importance. Then we utilized the remaining features in Apparent Cross-Validation, Leave-One-Out Cross-Validation, 3-Fold Random Subsampling Cross-Validation, and Repeated 3-Fold Cross-Validation to valuate predictive performance of the current random forest model (simulation 200 times for 3-Fold Random Subsampling Cross-Validation and Repeated 3-Fold Cross-Validation). Predictive accuracy (%), related sensitivity, specificity, positive predictive value (PPV), and negative predictive value (NPV) were criteria of model selection. The entire procedure (random forest, variable clustering; cross-validations) was iterated until the removal of any additional features resulted in the predictive ability of the model to consistently and dramatically deteriorated. The final model had the best predictive ability with the fewest features and was composed of glycans (features) from four of the IgG subtypes. These were A2G2F and A2BG0F on IgG, A2G1F and A2G2S1 on IgG1, A2G1F and A2G0F on IgG2 and A2BG1F on IgG3.

Finally, we evaluated our final model’s discriminating ability between the following groups: no fibrosis vs moderate fibrosis, moderate vs later fibrosis, and no fibrosis vs later fibrosis. Four Cross-Validations listed above was applied and the corresponding AUCs (area under the curve), specificities, sensitivities, PPVs, and NPVs were derived.

More detail on feature selection, cross validation and model development can be found in the **Supplementary Evidence File**.

## Results

**Figure 1:**
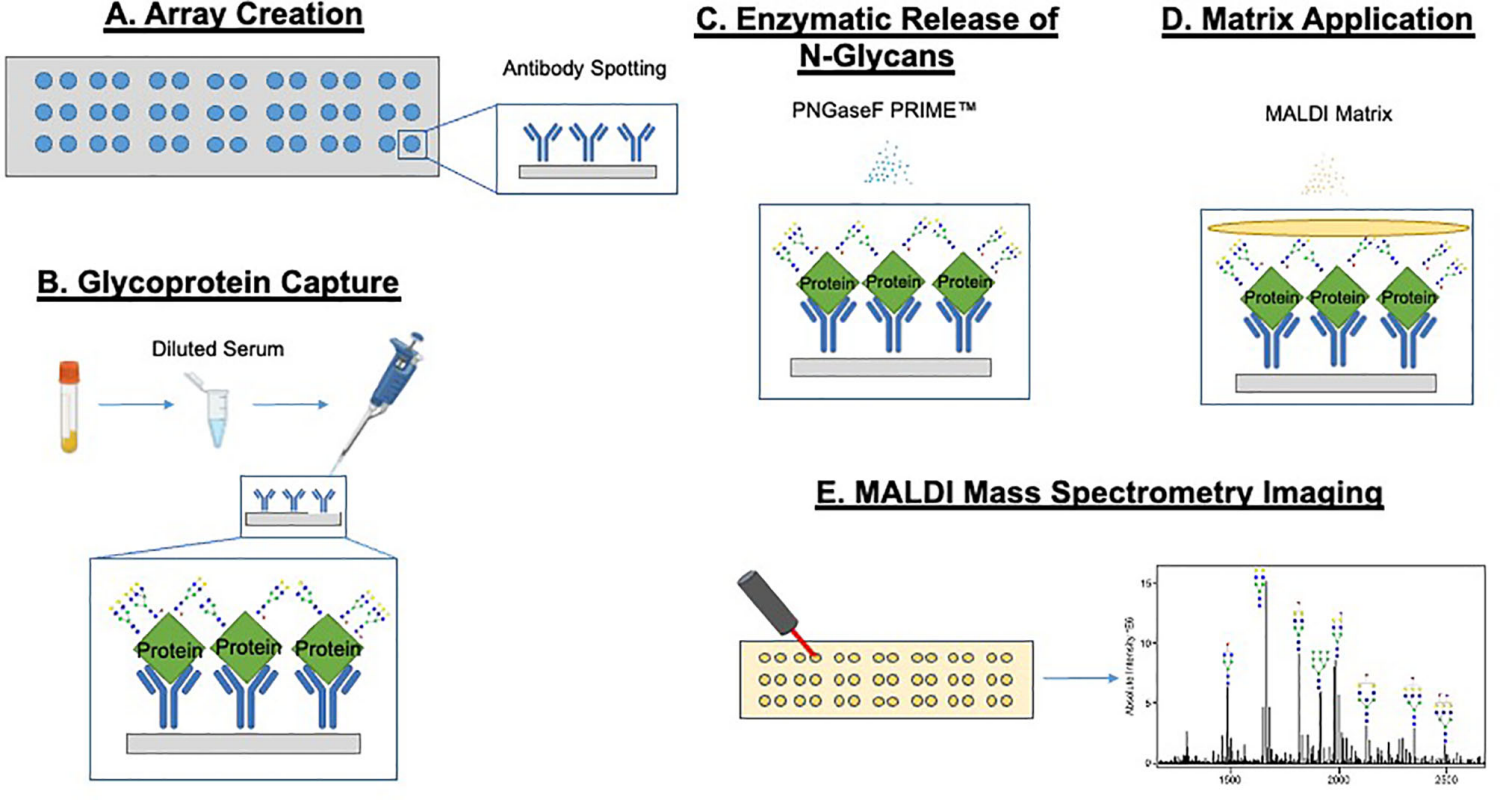
GlycoTyper workflow for the glycan analysis of antibody captured glycoproteins. The first step is the creation of the antibody array using the desired antibodies immobilized on nitrocellulose coated slides **(A)**. Subsequently, the antibody array is incubated with either diluted serum or any other protein mixture **(B)**. Slides are washed after protein capture using a mass spectrometry compatible detergent before glycan are released by application of a thin coating of recombinant PNGase F to the slide **(C)**. MALDI matrix is applied to the tissue and the slide imaged using a MALDI-Mass spectrometer **(D, E)**.

The basic GlycoTyper workflow for the glycan analysis of antibody captured glycoproteins is shown in **Figure 1**. Briefly, the first step is the creation of the antibody array using the desired antibodies immobilized on nitrocellulose coated slides (**Figure 1A**). Subsequently, the antibody array is incubated with either diluted serum or pure protein as shown in **Figure 1B**. Slides are washed after protein capture using a mass spectrometry compatible detergent before glycans are released by application of a thin coating of recombinant PNGase F PRIME™ to the slide (**Figure 1C**). This step is identical to what is performed in tissue glycan imaging (35, 42). Lastly, MALDI matrix is applied to the slide and is imaged using a MALDI Mass Spectrometer (**Figures 1D, E**). Importantly, the spatial localization provided by the IMS data link the detected glycans to their corresponding captured glycoproteins.

**Figure 2:**
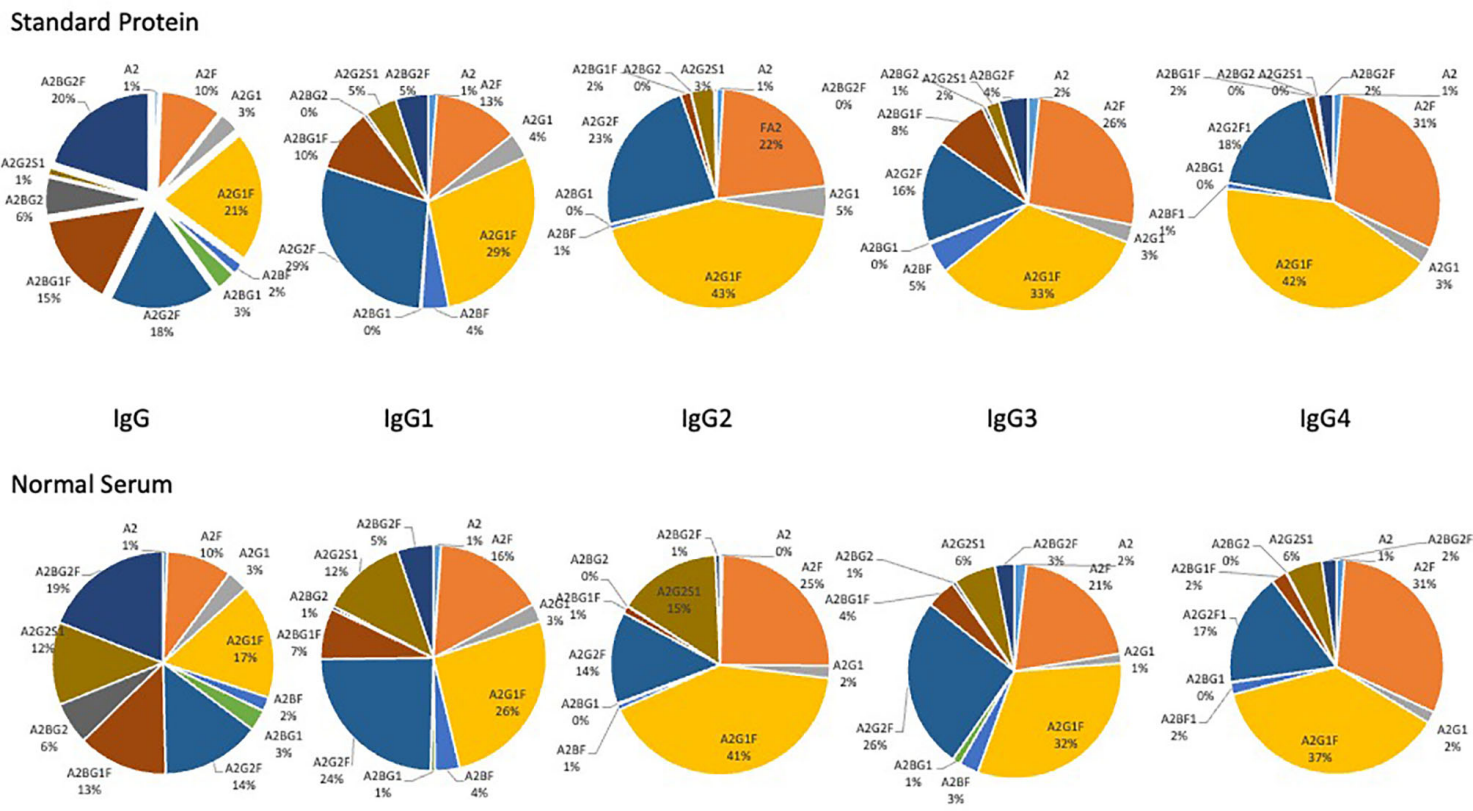
Glycan analysis of antibody captured IgG, IgG1, IgG2, IgG3 and IgG4 from either purchased purified protein (top) or from normal human serum (bottom). Glycans are presented as a function of the total glycan profile. For glycans, the Oxford notation is based on building up N-glycan structures and it can be used to denote all glycans (51). All N-glycans have two core GlcNAcs and three mannose residues that make up the trimannosyl core; F indicates a core fucose; Ax, where x- number of antenna (GlcNAc) on the trimannosyl core; Gx, where x- number of linked galactose on antenna; Sx, where x- number of sialic acids linked to galactose.

In initial studies, we examined the glycans on IgG, IgG1, IgG2, IgG3 and IgG4 captured *via* antibody from pure proteins spiked into PBS or from healthy serum diluted in PBS. As **Figure 2** shows for all proteins, the glycan observed from the pure protein was similar to that observed from captured human serum, with little difference in the glycosylation observed on the pure protein and that observed from the serum captured protein (with a single exception). The major exception was a glycan at m/z 1976.7822, which matches by accurate mass to a bi-antennary glycan with a single sialic acid (A2G2S1). In all cases, the level of this glycan was lower in the pure protein than that observed in human serum. In addition, a glycan at a glycan at m/z 1809.73273, which matches by accurate mass to a bi-antennary glycan with a single fucose residue was also varied in IgG2 and IgG3. **Supplementary Table S1** shows all the glycans found associated with IgG, IgG1, IgG2, IgG3 or IgG4 and **Supplementary Figure S1** shows a column chart of the data in **Figure 1**.

The major glycan observed for each immunoglobulin subtype was a fucosylated biantennary glycan with single or double galactose residues, with or without a bisecting N-acetyl glucosamine (**Figure 2**). It is noted that the glycan profile observed from both samples was consistent with the glycan profile observed by other methods for these proteins (16, 53–59).

### Simultaneous Analysis of the Glycans on IgG, IgG1, IgG2, IgG3 and IgG4 in Patients With Liver Fibrosis

**Figure 3:**
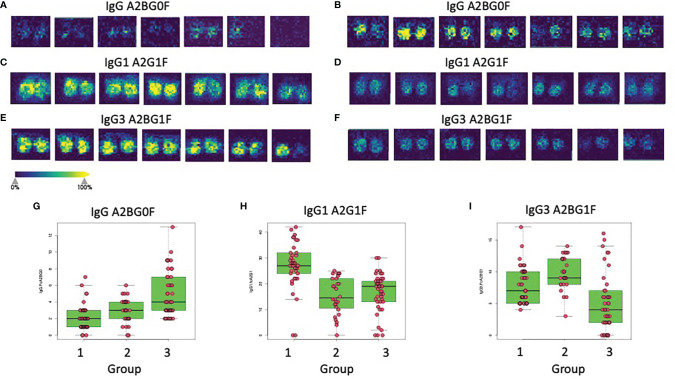
Glycan alterations on immunoglobulins correlate with the level of liver fibrosis. Panels A and B show the imaging analysis for the A2BG0F glycan on IgG from either Group 1 patients **(A)** or Group 3 patients **(B)**. **(C, D)** show the imaging analysis for the A2G1F glycan on IgG1 from either Group 1 patients **(C)** or Group 2 patients **(D)**. **(E, F)** show the imaging analysis for the A2BG1F glycan on IgG3 from either Group 1 patients **(E)** or Group 3 patients **(F)**. Panels G, H, and I show box and whiskers plots of the IgG A2BG0F glycan **(G)**, the IgG1 A2G1F glycan **(H)**, or the IgG3 A2BG1F **(I)** glycan in all patients from all three groups.

As previously reported, the glycosylation of IgG is altered with the development of liver fibrosis and cirrhosis and therefore can be used as a non-invasive measure of liver disease (11, 16, 18, 20, 30). Thus, the GlycoTyper method described in **Figure 1** was used for the analysis of a cohort of patients with biopsy confirmed fibrosis (**Table 1**). In total, 38 healthy individuals with no evidence of liver disease, 3 patients with stage 0 fibrosis (METAVIR), 10 patients with stage 1 fibrosis (METAVIR), 18 patients with stage 2 fibrosis (METAVIR), 6 patients with stage 3 fibrosis (METAVIR) and 37 patients with stage 4 fibrosis (METAVIR) were analyzed using this method. Because of the limited patient number, we grouped samples into three categories: i) those who are healthy with no fibrosis (healthy & METAVIR stage 0; n=41), ii) those with early to moderate fibrosis (METAVIR stages 1&2, n=28), and iii) those with severe fibrosis and cirrhosis (METAVIR stage 3&4; n=43). **Table 2** lists those glycans that were statistically different (p<0.05) between the three patient groups. Glycans are classified as those that were altered in early-stage fibrosis only (as compared to healthy or stage 0 fibrosis) and do not alter further with fibrosis progression, glycans which alter only with the progression to significant fibrosis, and those glycans that are altered with both the development of early fibrosis and again with more significant fibrosis. **Figure 3** shows examples of three glycans that were altered on IgG, IgG1 and IgG3. One of the glycans with the greatest changes included a core fucosylated agalactosylated bi-antennary glycan with a bisecting N-acetyl glucosamine (A2BG0F, **Figures 3A, B**). As **Figure 3A** shows, this glycan was found at low levels in either healthy patients or patients with stage 0 fibrosis but increased dramatically in those with cirrhosis (**Figure 3B**). A2BG0F was found to increase in abundance in those patients with significant fibrosis/cirrhosis (Group 2), on both total IgG (**Figures 3A, B**) and to a lesser degree IgG1 and IgG3 (data not shown). In contrast, the A2G1F, a core fucosylated bi-antennary glycan with a single galactose residue found on IgG1, decreased in those patients with fibrosis (**Figures 3C, D**). Similarly, A2BG1F, a core fucosylated bi-antennary glycan with a single galactose residue and a bisecting N-acetyl glucosamine, found on IgG3 also decreased in those patients with fibrosis (**Figures 3E, F**). As **Figures 3E, F** demonstrate, this glycan was found at high levels in either healthy patients or patients with stage 0 fibrosis but was reduced dramatically in those with cirrhosis (**Figure 3F**).

**Table 2:** Glycans that differentiate either early fibrosis from healthy, late fibrosis from early fibrosis or both.

Early	Late	Early and Later
G4.A2G0F	G4.A2BG2F	G4.A2BG0F
G4.A2G1	G.A2BG2F	G.A2G0F
G4.A2G1F	G1.A2G0F	G.A2BG0F
G4.A2G2F	G3.A2G2F	G.A2BG2F
G. A2BG0F		G1.A2BG0F
G1.A2G2F		G3.A2BG0F
G2.A2G0F		
G2.A2G0F		
G2.A2G1		
G2.A2G1F		
G2.A2G2F		
G2.A2BG1F		
G2.A2BG2F		
G3.A2G0F		
G3.A2G1F		
G3.A2G2F		

For glycan names, the Oxford notation is used (51). All N-glycans have two core GlcNAcs and three mannose residues that make up the trimannosyl core; F indicates a core fucose; Ax, where x- number of antenna (GlcNAc) on the trimannosyl core; Gx, where x- number of linked galactose on antenna; Sx, where x- number of sialic acids linked to galactose. Using the G4.A2BG0F glycan as an example, G4 refers to IgG4, A2 indicates a bi-antennary glycan, the G0 indicates zero galactose residues, B represents the presence of a bisecting N-acetylglucosamine (GlcNAc), and the F indicated the presence of a fucose.

The expression levels of both of these glycans also varied within the larger patient groups. In the case of IgG, the A2BG0F glycan increased from Group 1 to Group 2 and was the highest in Group 3, showing increased levels with increased fibrosis (**Figure 3G**). In contrast, IgG1 associated A2G1F glycan was reduced in patients with both limited and severe fibrosis and did not correlate with the state of fibrosis (**Figure 3H**). Other glycans such as the A2BG1F glycan on IgG3, was only reduced in patients with more significant fibrosis (Group 3, **Figure 3I**). A complete list of glycans that were altered in both those with mild to moderate fibrosis and those glycans altered with more significant fibrosis are shown in **Table 2**.

### Development of a Model to Determine the Level of Liver Fibrosis in Individuals Based Upon the IgG Glycan Profile

**Figure 4:**
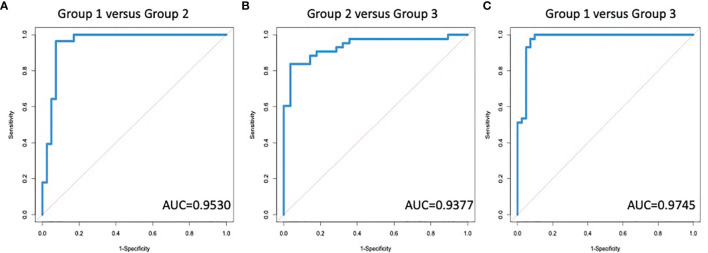
A Diagnostic algorithm based upon IgG glycans can identify the degree of fibrosis in patients with liver disease. AUROC for the differentiation of **(A)** group 1 (no fibrosis) versus group 2 (early to moderate fibrosis); **(B)** group 2 versus group 3 (significant fibrosis or cirrhosis); or **(C)** group 1 versus group 3.

Based on our previous algorithm development work for the early detection of HCC (34, 60–62), we developed a random forest algorithm that could be used to differentiate individuals with early fibrosis (Group 2) from healthy controls (Group 1) as well as identify late fibrosis patients (Group 3) from those with early fibrosis (Group 2). **Supplementary Table S2** shows the glycans that were found to be associated with IgG, IgG1, IgG2, IgG3 or IgG4 in all patient groups along with the results following ANOVA analysis. Although there were 50 glycans on the five subtypes of IgG that were altered with the development of liver fibrosis, many of these were associated and thus, only seven glycans on four glycoproteins were used to build and test the model. These are shown in **Supplementary Tables S3, S4** and include, A2G2F and A2BG0F on IgG, A2G1F and A2G2S1 on IgG1, A2G1F and A2G0F on IgG2 and A2BG1F on IgG3. **Figure 4A** shows the AUC (area under the curve) of the model following leave one out cross validation (LOOCV) when differentiating Group 1 (healthy and stage 0) from those with early or moderate fibrosis (Group 2; METAVIR stage 1 or 2). The AUC was 0.9530 (95%CI: 0.9008-1.0) for differentiating these two groups with a sensitivity of 94% at 90% specificity. Similar results were obtained using three-fold cross validation (3CV) (data not shown). When we compared those with early to moderate fibrosis (stage 1&2) to those with severe fibrosis or cirrhosis (stage 3 & 4), the AUC was 0.9377 (95%CI: 0.8819-0.9935) with a sensitivity of 84% at 90% specificity (**Figure 4B**). A further comparison was made to demonstrate that the AUC for differentiating those with no liver disease from those with significant fibrosis/cirrhosis (**Figure 4C**) and in this case, the AUC was 0.9745 (95%CI: 0.9415-1) with 98% sensitivity at 90% specificity. The sensitivity, specificity, positive and negative predictive values of the assay for differentiating the different groups are shown in **Supplementary Tables S5–S7**. In addition, a comparison between our assay and other noninvasive assays for the detection of liver fibrosis is shown in **Supplementary Table S8**. It is noted that in **Supplementary Table S8** the assay performance for the detection of ≥2 fibrosis or ≥4 fibrosis is compared, as these are the data most often presented for other markers.

We also examined the ability of these glycans (**Supplementary Figure S1**) to differentiate different groups of patients, specifically, those with no or early fibrosis (healthy and stage 0&1) from those with moderate to significant fibrosis (stage 2&3) and those with cirrhosis (stage 4) from those with moderate to significant fibrosis (stage 2&3). The AUC was 0.8464 (95%CI: 0.7576- 0.9352) for differentiating those with no or early fibrosis (healthy versus stage 0&1) from those with moderate to significant fibrosis (stage 2&3) but 0.9673 (95%CI: 0.93-1.0) when differentiating cirrhosis from moderate to significant fibrosis. These findings highlight that changes in IgG glycosylation can be observed *via* the GlycoTyper method and used to determine the level of liver fibrosis in individuals with liver disease.

## Discussion

The GlycoTyper is an imaging mass spectrometry platform for the multiplexed detection of N-glycans from individual glycoproteins. This method is suitable for the analysis of immunoglobulin molecules from a wide variety of biological samples. The development of this technique was based on a well-established protocol for enzymatic release of N-glycans from tissue sections for MALDI IMS (63). In this platform, antibodies are essential for the specific capture of glycoprotein targets from a complex biological mixture, similar to an ELISA. However, unlike an ELISA, no secondary antibody or lectin is needed for this methodology as mass spectrometry provides sensitive and molecular specific detection of distinct N-glycans. Antibody capture also negates the need for sample cleanup prior to MS analysis, which can be extensive (64).

We applied the GlycoTyper platform to the analysis of IgG glycans from patients with liver disease. As stated, changes in the glycosylation of immunoglobulins have been associated with liver fibrosis and cirrhosis previously and in many cases, those changes were detected with lectins or other indirect or laborious methods (16, 56). The results presented here show that these changes can be observed *via* the GlycoTyper method and furthermore, that these can be used to determine the level of liver fibrosis in individuals with liver disease. One of the major changes observed was an increase in the level of IgG associated glycans devoid of terminal galactose residues (**Figure 3**). This is a change that has been observed before and exploited with lectins (16). However, this was only one of numerous subtype specific glycan changes that were observed (**Figure 3**). These subtype specific glycan changes were used to create a diagnostic algorithm that could classify the level of fibrosis in individuals with high accuracy and specificity. It is noted that other glycoproteins have altered glycans in liver fibrosis and the methods described here could easily be applied to the analysis of those glycoproteins as well (65–69).

Our study was limited in part by the relatively small number of patients with histologically proven early stage liver disease (**Table 1**). This required clustering multiple patient groups together for further analysis. Consequently, patients were grouped into those with no fibrosis (Group 1), those with early to moderate fibrosis (Group 2) or late-stage fibrosis or cirrhosis (Group 3). We also attempted to classify patients into those with no to early fibrosis (healthy, stage 0&1), moderate to severe fibrosis (stage 2&3) and cirrhosis (stage 4). However, as shown in **Supplementary Figure S2**, diagnostic performance was poor in differentiating those with zero to early fibrosis from those with moderate to severe fibrosis. This was primarily the result of the stage 1 patients having an immunoglobulin glycan profile that was more similar to stage 2 patients than to stage 0 or healthy patients. This is an important point and clearly indicates that the changes in glycosylation observed on immunoglobulins occurs early in fibrosis development. It is also noted that excellent discriminatory ability was observed between those with moderate to severe fibrosis and cirrhosis. However, this is most likely driven by the small number of patients with severe fibrosis (n=6) and the large glycan difference observed in patients with cirrhosis and moderate fibrosis. Larger, more diverse studies that include patients with different types of clinical liver diseases and a direct comparison to other non-invasive tests for cirrhosis required (3, 11, 70–73)

In conclusion, this study establishes a clinical rationale for integrating direct analysis of IgG glycans for the active management of people with liver disease. The diagnostic performance of this method was dramatically greater than other non-invasive tests for liver fibrosis (**Supplementary Table S8**), such as FIB4, APRI and even tests that look at IgG glycans (11, 16, 74). One reason for this is the ability to not just look at total IgG but also the specific IgG sub-classes, which has not been performed before, as such studies were not technically feasible for large patient cohorts. Thus, the methods described here are not only useful as a research tool for the analysis of protein specific glycosylation but also represents a new diagnostic platform that will allow for the true diagnostic potential of N-linked glycans to be developed.

## Data Availability Statement

The original contributions presented in the study are included in the article/**Supplementary Material**. Further inquiries can be directed to the corresponding author.

## Ethics Statement

The studies involving human participants was reviewed and approved by the University of South California at San Diego and Medical University of South Carolina Review Board. The patients/participants provided their written informed consent to participate in research studies.

## Author Contributions

DS was involved in performance of assay, assay design, data analysis and writing of manuscript. MW was responsible of statistics and algorithm development. SG and SP were involved in performance of assay. AB was responsible for experimental design and assay development. PA, RD, and SC were involved in conceptualization of assay, data analysis and writing of manuscript. YK and DR provided samples for patient analysis, data analysis, editing of manuscript. AM was involved with conceptualization of assay design, data analysis and writing of manuscript. All authors contributed to the article and approved the submitted version.

## Funding

NIH/NIDDK: R41 DK124058. The goal is to develop a mass spectrometry based method for the detection of liver fibrosis. NIH/NCI: U01 CA242096. The goals of the grant are to develop rapid glycan analysis workflows using basic MALDI-TOF MS instrumentation common to most institutions. Slide-based analysis of N-glycans from immunoglobulin classes, immune cells and cells grown in culture on slides is emphasized. NIH/NCI: R01CA237659. The goal of this grant is to develop and validate a promising biomarker for the early detection of hepatocellular carcinoma. NIH/NCI: U01 CA226052. The goals are to determine the genetic basis of alterations in N-linked glycosylation observed (fucose or tetra-antennary glycan) in liver cancer typed by genetic classifications, and determine serum glycoproteins that directly reflect these changes for use as cancer biomarkers.

## Conflict of Interest

DS, SC, and SP all work for GlycoPath Inc, who have licensed technology from AM, PA, and RD for the glycan analysis of antibody captured glycoproteins.

The remaining authors declare that the research was conducted in the absence of any commercial or financial relationships that could be construed as a potential conflict of interest.

## Publisher’s Note

All claims expressed in this article are solely those of the authors and do not necessarily represent those of their affiliated organizations, or those of the publisher, the editors and the reviewers. Any product that may be evaluated in this article, or claim that may be made by its manufacturer, is not guaranteed or endorsed by the publisher.
