# Supplementary material for: GlycoFibroTyper: A Novel Method for the Glycan Analysis of IgG and the Development of a Biomarker Signature of Liver Fibrosis
Source: Front Immunol. 2022 Feb 7;13:797460. doi: 10.3389/fimmu.2022.797460 (PMC8858972; doi:10.3389/fimmu.2022.797460)
Supplement: Supplementary file 1 [file DataSheet_1.docx]

**Supplementary Table S1: Glycans identified as associated with IgG, IgG1, IgG2, IgG3 or IgG4**

For glycan names, the Oxford nomenclature is used. All N-glycans have two core GlcNAcs and three mannose residues that make up the trimannosyl core; F indicates a core fucose; Ax, where x- number of antenna (GlcNAc) on the trimannosyl core; Gx, where x- number of linked galactose on antenna; Sx, where x- number of sialic acids linked to galactose. In the case of the glycan nomenclature, using the G4.A2BG0F1 glycan. G4 refers to IgG4, A2 indicates a bi-antennary glycan, the G0 indicates zero galactose residues, B represents the presence of a bisecting N-acetylglucosamine (GlcNAc), and the F indicated the presence of a fucose.

**Supplementary Table S2:** ANOVA test for each glycan on each glycoprotein from the three groups.

|  | **Glycan** | **p-value** | **selected when p<0.01** |
| --- | --- | --- | --- |
| 1 | IgG.A2G0F | 4.64E-07 | selected |
| 2 | IgG.A2G1 | 2.34E-01 | not selected |
| 3 | IgG.A2G1F | 3.15E-07 | selected |
| 4 | IgG.A2BG0F | 6.00E-08 | selected |
| 5 | IgG.A2BG1 | 9.33E-03 | selected |
| 6 | IgG.A2G2F | 6.48E-07 | selected |
| 7 | IgG.A2BG1F | 5.60E-01 | not selected |
| 8 | IgG.A2BG2 | 2.80E-01 | not selected |
| 9 | IgG.A2G2S1 | 4.50E-07 | selected |
| 10 | IgG.A2BG2F | 2.32E-04 | selected |
| 11 | IgG1.A2G0F | 3.59E-01 | not selected |
| 12 | IgG1.A2G1 | 4.38E-02 | not selected |
| 13 | IgG1.A2G1F | 3.19E-10 | selected |
| 14 | IgG1.A2BG0F | 1.63E-05 | selected |
| 15 | IgG1.A2BG1 | 6.01E-04 | selected |
| 16 | IgG1.A2G2F | 4.53E-05 | selected |
| 17 | IgG1.A2BG1F | 1.42E-05 | selected |
| 18 | IgG1.A2BG2 | 7.47E-02 | not selected |
| 19 | IgG1.A2G2S1 | 5.01E-06 | selected |
| 20 | IgG1.A2BG2F | 7.28E-06 | selected |
| 21 | IgG2.A2G0F | 2.75E-07 | selected |
| 22 | IgG2.A2G1 | 5.40E-02 | not selected |
| 23 | IgG2.A2G1F | 4.26E-03 | selected |
| 24 | IgG2.A2G2 | 2.95E-01 | not selected |
| 25 | IgG2.A2BG0F | 4.39E-04 | selected |
| 26 | IgG2.A2BG1 | 6.90E-08 | selected |
| 27 | IgG2.A2G2F | 8.94E-01 | not selected |
| 28 | IgG2.A2BG1F | 1.36E-05 | selected |
| 29 | IgG2.A2BG2 | 8.78E-08 | selected |
| 30 | IgG2.A2G2S1 | 5.14E-04 | selected |
| 31 | IgG3.A2G0F | 2.02E-03 | selected |
| 32 | IgG3.A2G1 | 1.94E-07 | selected |
| 33 | IgG3.A2G1F | 1.26E-02 | not selected |
| 34 | IgG3.A2BG0F | 9.50E-01 | not selected |
| 35 | IgG3.A2BG1 | 4.90E-04 | selected |
| 36 | IgG3.A2G2F | 5.28E-01 | not selected |
| 37 | IgG3.A2BG1F | 6.68E-06 | selected |
| 38 | IgG3.A2BG2 | 4.81E-04 | selected |
| 39 | IgG3.A2G2S | 3.14E-04 | selected |
| 40 | IgG3.A2BG2F | 8.62E-04 | selected |
| 41 | IgG4.A2G0F | 1.97E-03 | selected |
| 42 | IgG4.A2G1 | 6.81E-01 | not selected |
| 43 | IgG4.A2G1F | 3.57E-02 | not selected |
| 44 | IgG4.A2BG0F | 3.44E-04 | selected |
| 45 | IgG4.A2BG1 | 3.32E-04 | selected |
| 46 | IgG4.A2G2F | 5.71E-03 | selected |
| 47 | IgG4.A2BG1F | 4.82E-01 | not selected |
| 48 | IgG4.A2BG2 | 7.94E-01 | not selected |
| 49 | IgG4.A2G2S1 | 2.78E-08 | selected |
| 50 | IgG4,A2BG2F | 5.95E-02 | not selected |

**Supplementary Table S3. The seven glycans from three proteins that were chosen for inclusion into the final model.**

| **Glycan** |
| --- |
| IgG.A2G2F |
| IgG.A2BG0F |
| IgG1.A2G1F |
| IgG1.A2G2S1 |
| IgG2.A2G1F |
| IgG2.A2G0F |
| IgG3.A2BG1F |

**Supplementary Table S4. Relative importance of each glycan in final model of random forest.**

| Glycan name | Relative importance |
| --- | --- |
| IgG.A2G2F | 11.78 |
| IgG2.A2G0F | 6.57 |
| IgG1.A2G1F | 6.31 |
| IgG1.A2G2S1 | 4.44 |
| IgG3.A2BG1F | 2.79 |
| IgG2.A2G1F | 2.45 |
| IgG.A2BG0F | 1.38 |

**Table S5. Group 1 versus Group 2 Performance.**

| Sensitivity | 0.9268 |
| --- | --- |
| Specificity | 0.8929 |
| Pos Predictive Value | 0.9268 |
| Neg Predictive Value | 0.8929 |

Accuracy=0.9130, 95%CI: 0.8203-0.9674

AUC=0.9530, 95%CI: 0.9008-1

**Table S6. Group 2 versus 3 Performance.**

| Sensitivity | 0.8214 |
| --- | --- |
| Specificity | 0.8837 |
| Pos Predictive Value | 0.8214 |
| Neg Predictive Value | 0.8837 |

Accuracy=0.8592, 95%CI: 0.7562-0.9303

AUC=0.9377, 95%CI: 0.8819-0.9935

**Table S7. Group 1 Versus Group 3 Performance.**

| Sensitivity | 0.9268 |
| --- | --- |
| Specificity | 0.9767 |
| Pos Predictive Value | 0.9744 |
| Neg Predictive Value | 0.9333 |

Accuracy=0.9524, 95%CI: 0.8825-0.9869

AUC=0.9745, 95%CI: 0.9415-1

**Supplementary Table S8. Comparison between the GlycoFibroTest and other non-invasive tests.**

| **Test^1^** | **AUROC F>2^2^** | **AUROC F>4^3^** | **Ref^4^** |
| --- | --- | --- | --- |
| FIB4 | 0.70 | 0.84 | (Lombardi et al. 2015) |
| APRI | 0.82 | 0.92 | (Ichino et al. 2010) |
| FibroTest | 0.82-0.88 | 0.92 | (Lai and Afdhal 2019) |
| Forns Index | 0.78 | 0.88 | (Ichino, et al. 2010) |
| GlycoCirrhotest | 0.71 | 0.87 | (Vanderschaeghe et al. 2009) (Callewaert et al. 2004) |
| LRAGG | 0.92 | 0.93 | (Mehta et al. 2008) |
| GlycoFibroTest^5^ | 0.92 | 0.98 |  |

**(1)** Several of the common serum based non invasive tests for the detection of liver fibrosis. Fib4= the fibrosis 4 index is an algorithm that utilizes age, AST, ALT and platelets to determine degree of liver fibrosis. APRI= AST to platelet ratio to determine degree of liver fibrosis. FibroTest, (also called FibroSure) is an algorithm that uses the level of six serum proteins to to determine degree of liver fibrosis. The Forns Index = an algorithm that utilizes age, gamma-glutamyltransferase (GGT), cholesterol, and platelet count to determine degree of liver fibrosis. GlycoCirrhotest uses total serum glycan analysis to to determine degree of liver fibrosis. LRAGG utilizes the level of lectin reactive anti-alpha gal antibodies to determine degree of liver fibrosis. The GlycoFibroTest is as described in this manuscript. **(2)** The AUROC for each test in the detection of liver fibrosis at or above Metavir Stage 2. **(3)** The AUROC for each test in the detection of liver fibrosis at or above Metavir Stage 4**. (4)** Reference for the indicated AUC values. It is noted that for many of these tests, other references also exist but are not include for space considerations. (**5**). Data as presented in this manuscript from leave one out cross validation.

**Supplementary Figure S1: Bar Chart of Figure 1 highlighting the similarity in glycan profile of captured pure protein and serum captured protein.** Glycans are presented as a function of the total glycan profile. Serum and Protein glycan are indicated as in IgG4. For glycans, the Oxford notation is based on building up N-glycan structures and it can be used to denote all glycans (Harvey et al., 2009). All N-glycans have two core GlcNAcs and three mannose residues that make up the trimannosyl core; F indicates a core fucose; Ax, where x- number of antenna (GlcNAc) on the trimannosyl core; Gx, where x- number of linked galactose on antenna; Sx, where x- number of sialic acids linked to galactose.

**Supplementary Figure S2:** AUROC for the differentiation of (A) those with no or early fibrosis (healthy and stage 0&1) from those with moderate to significant fibrosis (stage 2&3); (B) those with moderate to significant fibrosis (stage 2&3) from those with cirrhosis (stage 4); or (C) the differentiation of those with cirrhosis (stage 4) from those who have no liver fibrosis. AUC values are indicated.

**Statistical Methods and Learning algorithms:**

1. ***Feature selection:***

***ANOVA and Post hoc test:***

Because the distribution of some features(glycans) deviated from Gaussian distribution and limited sample size, we applied a one-way nonparametric ANOVA test (Kruskal-Wallis ANOVA) to determine the how well each glycan could discriminate between the 3 groups. Subsequently, we applied a pairwise Wilcox test with Dunn-Bonferroni adjustment to check the statistical difference between each pair of stages. Glycan with no statistical difference among stages were removed from further feature selection(p>0.01).

*For example:*

IgG.A2BG0F is selected.

IgG.A2BG0F

ANOVA:

p-value=6.00E-08

Post hoc:

p-value matrix:

|  | stage0 | stage1 |
| --- | --- | --- |
| stage1 | 5.33E-02 | NA |
| stage2 | 7.53E-08 | 0.00439883 |

Trend test:

P=8.14E-09

IgG.A2BG2 was discarded.

IgG.A2BG2

ANOVA

p-value=2.80E-01

Post hoc test:

p-value matrix

|  | stage0 | stage1 |
| --- | --- | --- |
| stage1 | 0.2573479 | NA |
| stage2 | 0.8061084 | 1 |

Trend test:

P=0.116

*Procedure of random forest, variable clustering, validations.*

We applied random forest as an embedded approach of feature selection in this study.

We focused on features with high importance in random forest analysis, with cognizance of their relationship. That is, glycans with similar performance and contributions imply some underlying information without much extra information added, and highly correlated features causes potential statistical problem of “multicollinearity”.

In our study, we applied variable clustering to determine the most associated variables. In cases were glycans had high similarity of data structure, we only keep the one with relatively higher importance.

For example, cluster analysis below, IgG.A2GF and IgG2.A2BG1 were highly correlated.

The importance of IgG.A2G2F was 7.61 while the importance of IgG2.A2BG1 was 4.89; thus we discard the IgG2.A2BG1 glycan as it co similar but of less importance to the model.

Selected glycans were evaluated by 4 kinds of validations to check predictive performance. Predictive accuracies and related sensitivity, specificity, positive predictive value and negative predictive value were criteria of feature selection procedure.

Random forest analysis, variable clustering and validation form a feature selection procedure. This 3 step-procedure as a feature selection circle was kept running and after each iteration, a variable was removed. The model was stopped only when there was a substantial decrease of predictive performances. Thus, this method allowed for creation of a model with the least number of features.

To challenge the robustness of our selected features, we introduced some features from other methods into the model in an attempt to “disturb” our feature selection process.

We applied ordinal tree analysis and conditional tree analysis to produce some features which were not in selected by our procedure and incorporated them in our feature selection procedure. After couple rounds of our feature selection process, these “artificial” features were filtered out, validating the feature selectin process.

We also applied the final random forest model to evaluate its predictive ability between the groups. This was done through the comparison of one group with another group. In this study, ROC curves were constructed, corresponding accuracy, sensitivity, specificity, PPV, NPV were all considered.

1. *Cross-validations:*

For all models and testing, we utilized apparent validation and three cross-validations methods to determine the accuracy of classification and prediction of the random forest models in every stage of feature selection. The validations were:

- 1. *Apparent Validation*.
  2. *3-folds random subsampling Cross-Validation*.
  3. *Leave-one-out Cross-Validation(LOOCV)*.
  4. Repeated 3-folds Cross-Validation.

*Scatter plot of glycans selected in the final model.*

IgG.A2G2F

ANOVA:

p-value= 6.48E-07

IgG.A2G2

0 1

1 1.826518e-01 NA

2 5.530104e-10 1

IgG.A2BG0F

ANOVA:

p-value= 6.00E-08

IgG.A2BG0F

0 1

1 5.328304e-02 NA

2 7.526206e-08 0.004398829

IgG1.A2G1F

ANOVA:

p-value= 3.19E-10

IgG1.A2G1F

0 1

1 1.514234e-07 NA

2 2.529726e-08 1

IgG1.A2G1S1

ANOVA:

p-value= 5.01E-06

IgG1.A2G2S1

0 1

1 8.326046e-05 NA

2 2.763245e-06. 1

IgG2.A2G1F

ANOVA:

p-value= 4.26E-03

IgG2.A2G1F

0 1

1 0.45610228 NA

2 0.01889369 0.02044509

IgG2.A2G0F

ANOVA:

p-value=2.75E-07

IgG2.A2G0F1

0 1

1 1.923627e-03 NA

2 9.280236e-08 1

IgG3.A2BG1F

ANOVA:

p-value= 6.68E-06

IgG3. A2BG1F

0 1

1 0.017225518 NA

2 0.002346984 8.339116e-05

**References:**

Callewaert N, Van Vlierberghe H, Van Hecke A, Laroy W, Delanghe J, Contreras R. 2004. Noninvasive diagnosis of liver cirrhosis using DNA sequencer-based total serum protein glycomics. Nature medicine. Apr;10:429-434. Epub 2004/05/22.

Ichino N, Osakabe K, Nishikawa T, Sugiyama H, Kato M, Kitahara S, Hashimoto S, Kawabe N, Harata M, Nitta Y, et al. 2010. A new index for non-invasive assessment of liver fibrosis. World J Gastroenterol. Oct 14;16:4809-4816. Epub 2010/10/13.

Lai M, Afdhal NH. 2019. Liver Fibrosis Determination. Gastroenterol Clin North Am. Jun;48:281-289. Epub 2019/05/03.

Lombardi R, Buzzetti E, Roccarina D, Tsochatzis EA. 2015. Non-invasive assessment of liver fibrosis in patients with alcoholic liver disease. World J Gastroenterol. Oct 21;21:11044-11052. Epub 2015/10/27.

Mehta AS, Long RE, Comunale MA, Wang M, Rodemich L, Krakover J, Philip R, Marrero JA, Dwek RA, Block TM. 2008. Increased levels of galactose-deficient anti-Gal immunoglobulin G in the sera of hepatitis C virus-infected individuals with fibrosis and cirrhosis. Journal of virology. Feb;82:1259-1270. Epub 2007/11/30.

Vanderschaeghe D, Laroy W, Sablon E, Halfon P, Van Hecke A, Delanghe J, Callewaert N. 2009. GlycoFibroTest is a highly performant liver fibrosis biomarker derived from DNA sequencer-based serum protein glycomics. Mol Cell Proteomics. May;8:986-994. Epub 2009/02/03.
